# Supplementary material for: Engineering Pseudomonas putida KT2440 for chain length tailored free fatty acid and oleochemical production
Source: Commun Biol. 2022 Dec 12;5:1363. doi: 10.1038/s42003-022-04336-2 (PMC9744835; doi:10.1038/s42003-022-04336-2)
Supplement: Supplementary file 2 — Supplementary Information [file 42003_2022_4336_MOESM2_ESM.pdf]

Supplementary Information

Engineering *Pseudomonas putida* KT2440 for chain length tailored free fatty acid and oleochemical production

Authors & Affiliations

Luis E. Valencia [1,2,3], Matthew R. Incha[1,2,4], Matthias Schmidt[1,2,5], Allison N. Pearson[1,2,4], Mitchell G. Thompson[1,6], Jacob B. Roberts[1,2,3], Marina Mehling[1,2], Kevin Yin[1,2,4], Ning Sun[2,7], Asun Oka[2,7], Patrick M. Shih[1,2,4,6], Lars M. Blank[5], John Gladden[1,8], Jay D. Keasling[1,2,3,9-11*]

[1] Joint BioEnergy Institute, Emeryville, CA 94608, USA
[2] Biological Systems and Engineering Division, Lawrence Berkeley National Laboratory, Berkeley, CA 94720, USA
[3] Department of Bioengineering, University of California, Berkeley, CA 94720, USA
[4] Department of Plant and Microbial Biology, University of California, Berkeley, CA 94720, USA
[5] Institute of Applied Microbiology (iAMB), Aachen Biology and Biotechnology (ABBt), RWTH Aachen University, Aachen, Germany
[6] Environmental Genomics and Systems Biology Division, Lawrence Berkeley National Laboratory, Berkeley, California, USA
[7] Advanced Biofuels and Bioproducts Process Demonstration Unit, 5885 Hollis Street, Emeryville, California 94608, USA
[8] Biomanufacturing and Biomaterials Department, Sandia National Laboratories, Livermore, CA 94550, USA
[9] Department of Chemical & Biomolecular Engineering, University of California, Berkeley, CA 94720, USA
[10] Center for Biosustainability, Danish Technological University, Lyngby, Denmark
[11] Center for Synthetic Biochemistry, Institute of Synthetic Biology, Shenzhen Institutes of Advanced Technologies, Shenzhen, China

*Correspondence to keasling@berkeley.edu

Table of Contents

Supplementary Figure 1 - CoA Ligase Mutant RB-TnSeq Fitness Data; Page 3
Supplementary Figure 2 - Carbon source growth assays; Page 4
Supplementary Figure 3 - Isovalerate production; Page 5
Supplementary Figure 4 - Isopropyl myristate growth assays; Page 6
Supplementary Table 1 - FAME evaporation factors; Page 7
Supplementary Table 2. FFA titers in various *P. putida* strains after growth in 250 mL shake flask cultures; Page 8
Supplementary Table 3. FAME titers in various *P. putida* strains after growth in 250 mL shake flask cultures; Page 8
Supplementary Table 4. FFA yield; Page 9
Supplementary Table 5. Calculated FFA theoretical yield; Page 9
Supplementary Table 6. FAME yield; Page 9
Supplementary Table 7. Calculated FAME theoretical yield; Page 9

Supplementary Table 8. FFA production strategies and titers across different host organisms; Page 10
Supplementary Table 9. FAME production strategies and titers across different host organisms; Page 11
Supplementary Table 10. Strains used in this study; Page 12
Supplementary Table 11. Plasmids used in this study; Page 13
Supplementary References; Page 14

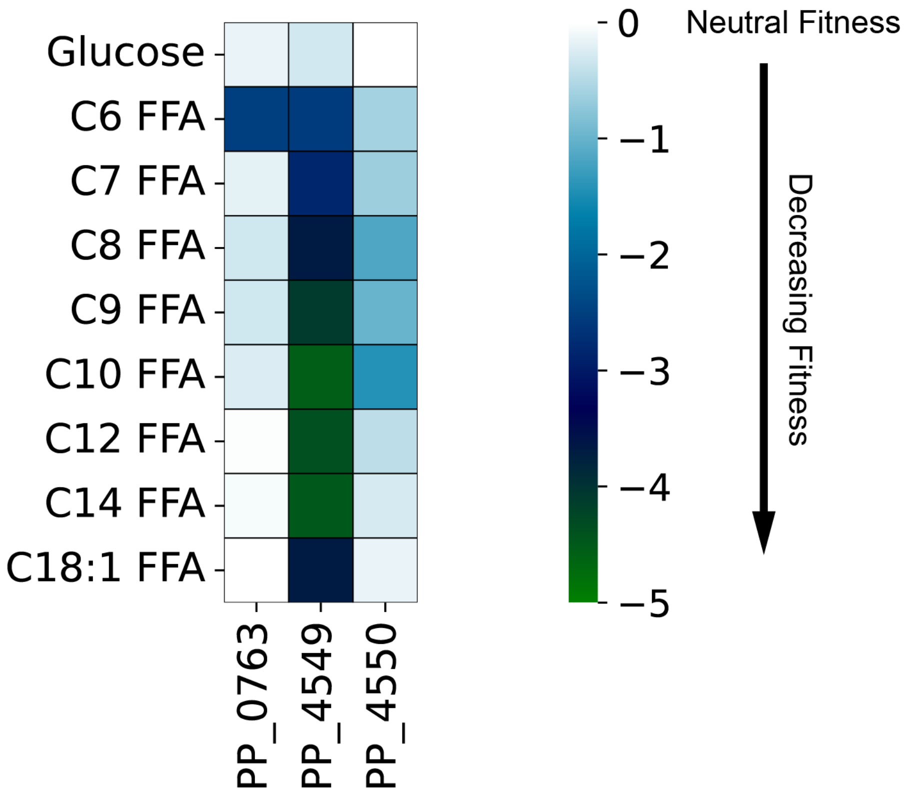

Supplementary Figure 1. CoA Ligase Mutant RB-TnSeq Fitness Data. Heat map showing fitness scores for various CoA ligase knockouts grown minimal media containing free fatty acids or glucose as a sole carbon source. The colors represent fitness scores, with white representing neutral fitness and green representing negative fitness.[1]

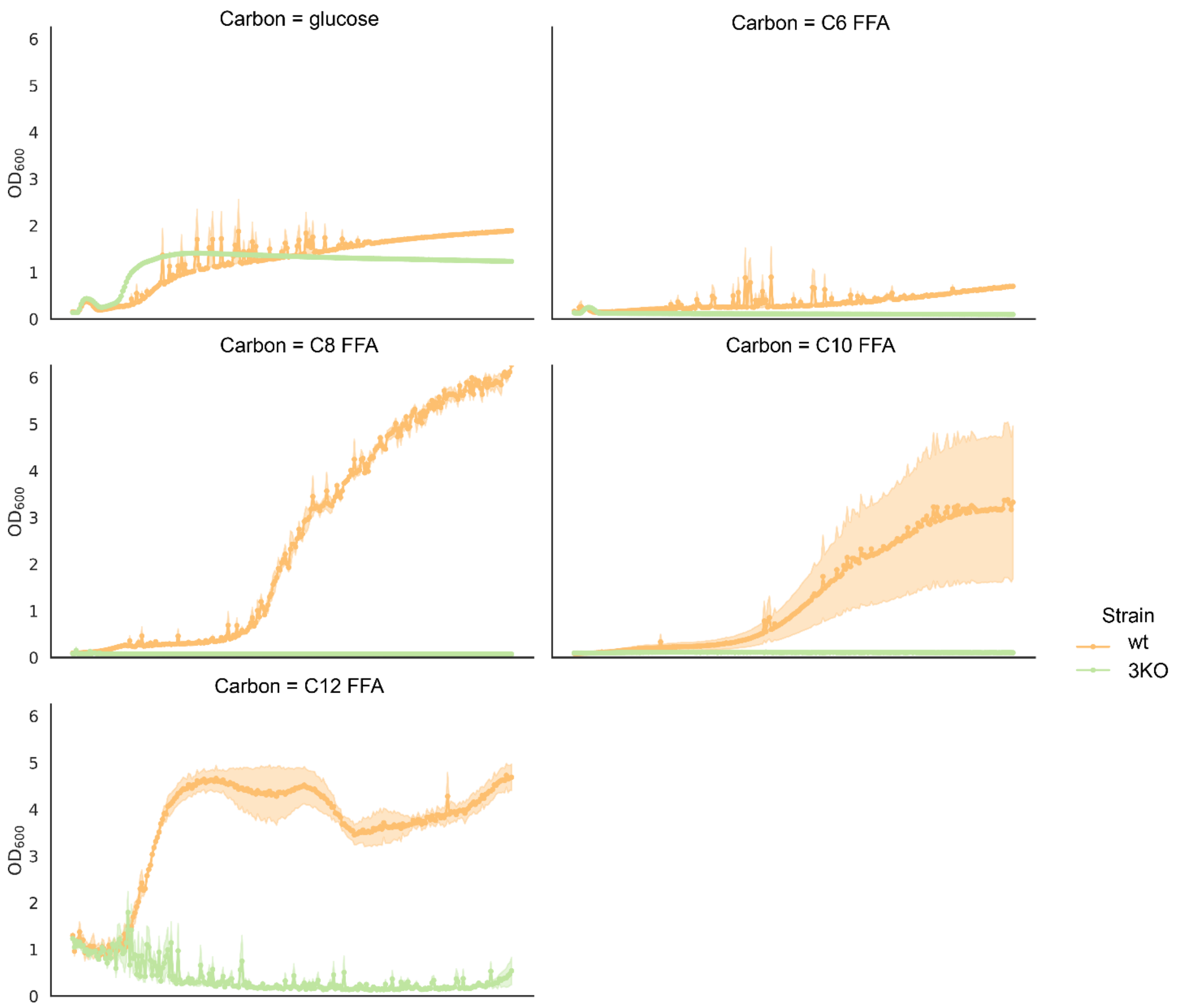

Supplementary Figure 2. Growth curves for wt and 3KO on glucose or medium-chain FFAs. The dark lines represent the mean of biologically independent samples (n=3), and the lighter shading shows the standard deviation.

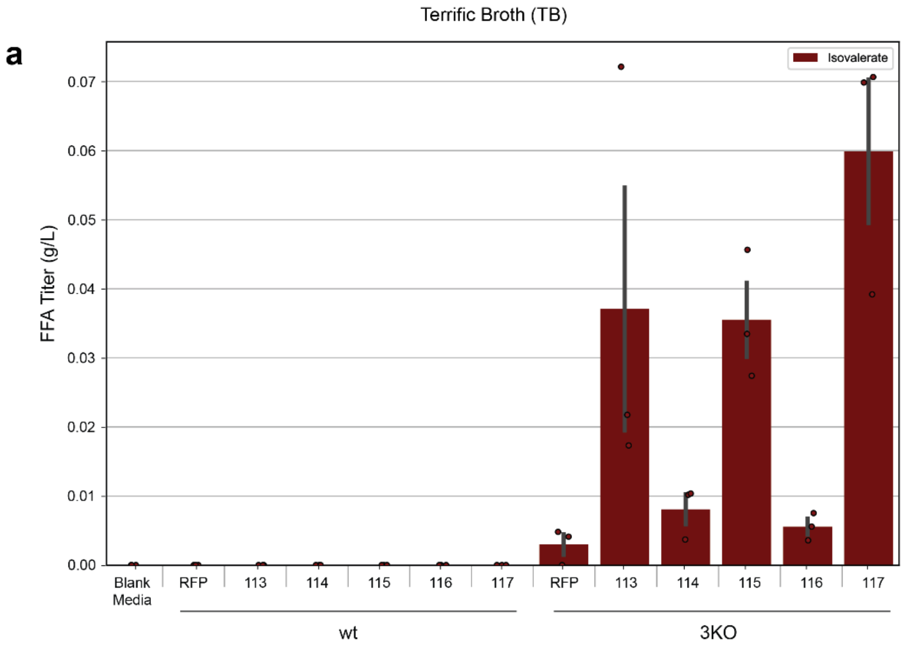

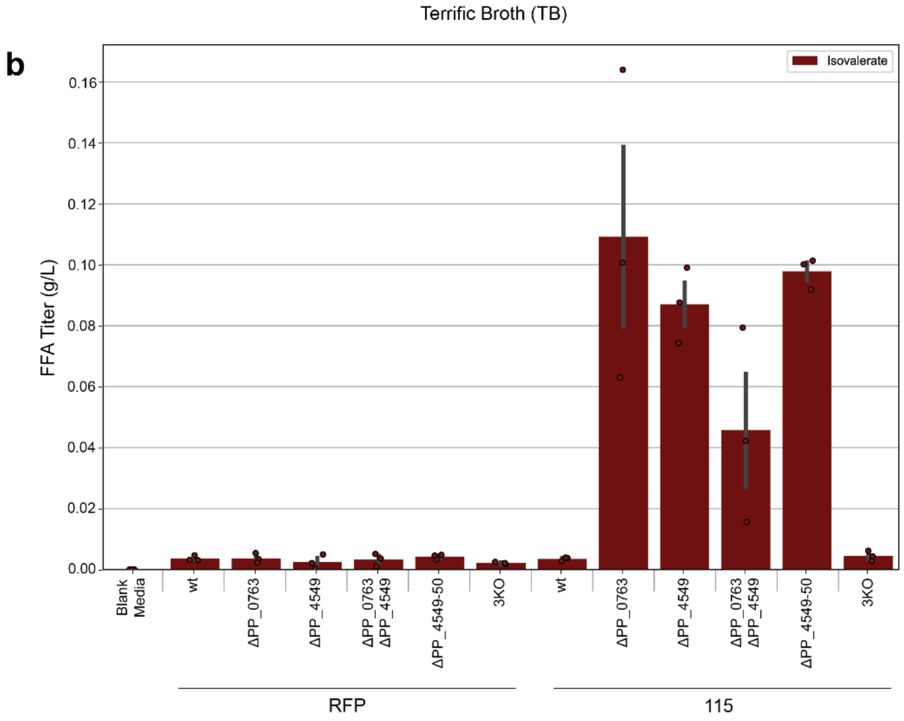

Supplementary Figure 3. Isovalerate production in various *P. putida* strains. a Isovalerate production in Terrific Broth across various ‘TesA variants in wt and 3KO background strains. **b** Isovalerate production across various *P. putida* background strains after overexpression of RFP or ‘TesA R3.M4. Values shown represent the mean of biologically independent samples (n = 3), and error bars show standard error of the mean.

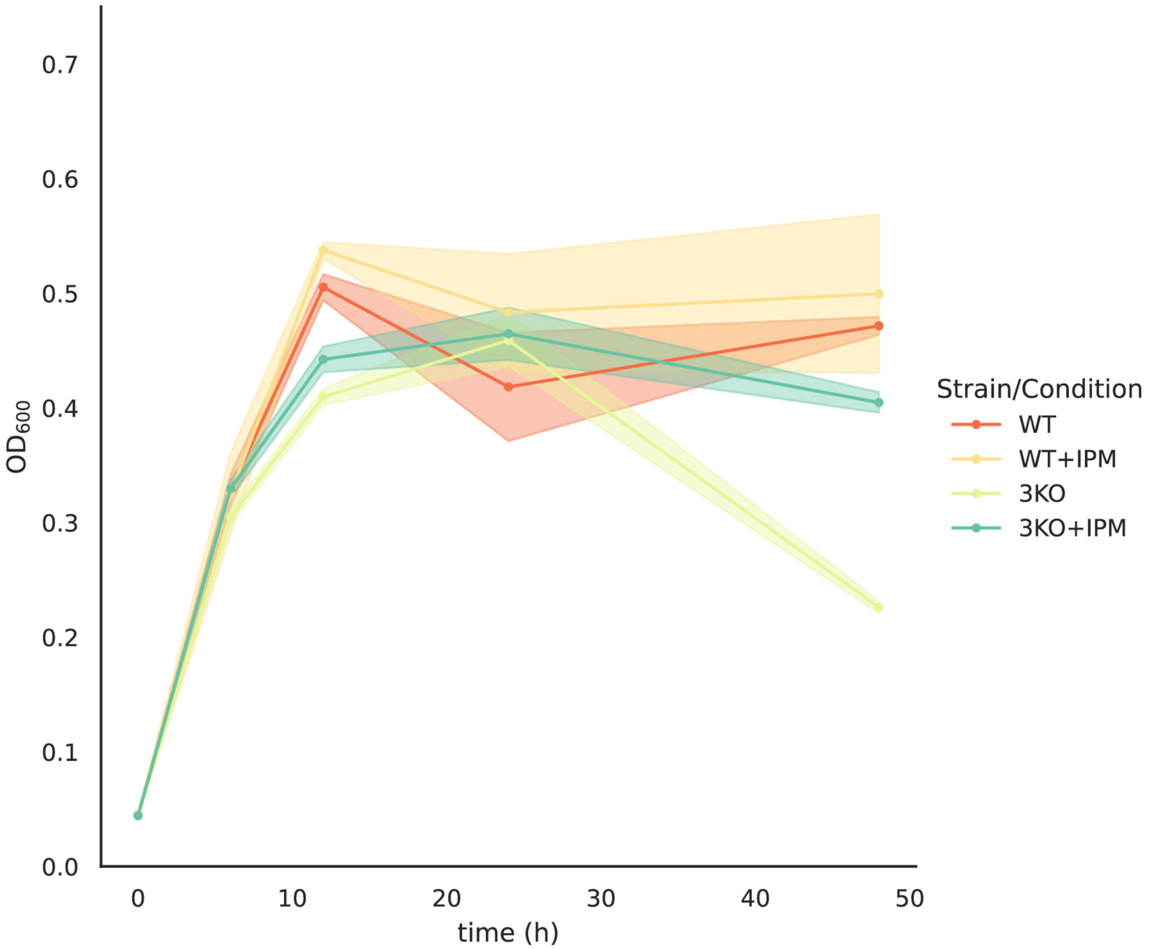

Supplementary Figure 4. Growth curves for strains grown in liquid culture (LB) with and without an isopropyl myristate overlay. The dark lines represent the mean of biologically independent samples (n=3), and the lighter shading shows the standard deviation.

Figure	Media	FAME	Evaporation Factor, E
4b	EZ Rich + 100 mM Glycerol	C6	2.060
		C8	1.135
		C10	1.030
		C12	0.988
4d	TB	C6	1.953
		C8	1.058
		C10	0.977
		C12	0.961
6b	EZ Rich + 100 mM Glycerol	C6	1.763
		C8	1.077
		C10	1.015
		C12	1.004
	TB	C6	1.945
		C8	1.108
		C10	0.993
		C12	0.970
	Diluted Sorghum Hydrolysate	C6	4.478
		C8	1.402
		C10	1.071
		C12	1.017

Supplementary Table 1. FAME evaporation factors.

Media	Strain	FFA Titer (mg/L)										Titer Fraction Medium-Chain
		C6	C8	C10	C12:1	C12	C14:1	C14	C16:1	C16	Total	
EZ Rich + 100mM Glycerol	Δ0763 Δ4549 + 115	21.0 ± 0.1	81.5 ± 0.5	5.3 ± 0.1	21.2 ± 0.2	21.7 ± 0.5	9.3 ± 0.4	1.2 ± 0.4	25.4 ± 0.6	2.7 ± 0.4	189.3 ± 3.0	0.80
	3KO + 115	11.9 ± 0.8	85.4 ± 5.1	5.0 ± 0.5	22.7 ± 1.6	27.3 ± 2.4	9.7 ± 1.2	14.5 ± 2.1	48.2 ± 1.1	34.3 ± 5.0	258.9 ± 19.8	0.59
	3KO + RFP	0.0 ± 0.0	0.0 ± 0.0	0.0 ± 0.0	0.0 ± 0.0	0.0 ± 0.0	0.0 ± 0.0	0.0 ± 0.0	33.5 ± 0.3	16.0 ± 2.6	49.5 ± 2.9	0.00
Terrific Broth	Δ0763 Δ4549 + 115	47.3 ± 5.2	122.3 ± 8.0	8.3 ± 1.1	19.1 ± 10.4	0.0 ± 0.0	0.0 ± 0.0	0.0 ± 0.0	0.0 ± 0.0	0.0 ± 0.0	197.0 ± 24.7	1.00
	3KO + 115	36.3 ± 5.1	253.6 ± 3.1	26.4 ± 0.9	84.3 ± 4.0	97.5 ± 6.6	30.3 ± 1.3	53.9 ± 3.3	50.1 ± 4.6	38.4 ± 2.3	670.9 ± 31.3	0.74
	3KO + RFP	0.0 ± 0.0	0.6 ± 0.0	0.0 ± 0.0	0.0 ± 0.0	0.7 ± 0.2	5.9 ± 1.0	11.8 ± 1.4	183.1 ± 1.8	395.1 ± 24.0	597.2 ± 28.4	0.00
Diluted Dry Sorghum Hydrolysate	Δ0763 Δ4549 + 115	43.0 ± 1.4	210.2 ± 4.0	26.2 ± 0.9	87.7 ± 1.7	79.8 ± 4.0	13.3 ± 4.7	46.6 ± 10.4	19.5 ± 4.6	35.0 ± 4.8	561.1 ± 36.5	0.80
	3KO + 115	1.4 ± 0.8	147.9 ± 1.7	13.7 ± 0.6	54.5 ± 0.8	75.4 ± 1.3	31.5 ± 0.4	60.7 ± 3.2	49.5 ± 3.6	51.5 ± 2.6	486.1 ± 15.0	0.60
	3KO + RFP	0.0 ± 0.0	10.4 ± 3.5	0.0 ± 0.0	0.0 ± 0.0	3.0 ± 0.5	3.6 ± 0.3	5.4 ± 0.5	16.7 ± 1.1	47.8 ± 2.3	86.9 ± 8.2	0.15

Supplementary Table 2. FFA titers in various *P. putida* strains after growth in 250 mL shake flask cultures. The fraction of the total titer that was composed of medium-chain FFAs is reported in the final column. Values shown represent the mean of biologically independent samples and the standard error of the mean (n = 3).

Media	Strain	FAME Titer (mg/L)					
		C6	C8	C10	C12:1	C12	Total
EZ Rich + 100mM Glycerol	Δ0763 Δ4549 + 125	1.0 ± 0.4	23.1 ± 3.4	0.8 ± 0.4	5.8 ± 0.3	0.0 ± 0.0	30.7 ± 4.5
	3KO + 125	1.7 ± 0.4	23.5 ± 5.9	0.0 ± 0.0	3.4 ± 0.5	0.0 ± 0.0	28.6 ± 6.8
Terrific Broth	Δ0763 Δ4549 + 125	25.8 ± 0.3	199.0 ± 10.7	22.4 ± 1.3	35.0 ± 0.9	20.1 ± 1.1	302.4 ± 14.2
	3KO + 125	25.0 ± 0.9	187.0 ± 13.0	15.6 ± 2.0	30.6 ± 2.4	20.3 ± 2.0	278.5 ± 20.2
Diluted Dry Sorghum Hydrolysate	Δ0763 Δ4549 + 125	11.7 ± 1.3	43.0 ± 6.0	2.1 ± 1.4	5.7 ± 0.8	1.0 ± 1.0	63.4 ± 10.5
	3KO + 125	27.0 ± 1.6	167.1 ± 10.8	8.9 ± 1.6	12.1 ± 0.8	8.9 ± 1.4	224.0 ± 16.1

Supplementary Table 3. FAME titers in various *P. putida* strains after growth in 250 mL shake flask cultures. Values shown represent the mean of biologically independent samples and the standard error of the mean (n = 3).

Media	Strain	FFA Yield (% of theoretical yield)								
		C6	C8	C10	C12:1	C12	C14:1	C14	C16:1	C16
EZ Rich + 100mM Glycerol	Δ0763 Δ4549 + 115	0.419	1.809	0.126	0.522	0.538	0.235	0.030	0.658	0.071
	3KO + 115	0.237	1.895	0.119	0.559	0.677	0.245	0.368	1.249	0.899
	3KO + RFP	0.000	0.000	0.000	0.000	0.000	0.000	0.000	0.868	0.419

Supplementary Table 4. FFA yield. FFA yield as a percent of maximum theoretical yield when grown on EZ Rich supplemented with 100 mM glycerol in 250 mL shake flask cultures.

	C6	C8	C10	C12:1	C12	C14:1	C14	C16:1	C16
FFA Theoretical yield (mol/mol of glycerol)	0.436	0.315	0.246	0.206	0.202	0.176	0.173	0.152	0.149
FFA Theoretical yield from 100mM glycerol (g/L)	5.016	4.506	4.217	4.063	4.031	3.963	3.938	3.858	3.817

Supplementary Table 5. Calculated FFA theoretical yield. Calculated FFA maximum theoretical yield from 100 mM glycerol.

Media	Strain	FAME Yield (% of theoretical yield)				
		C6	C8	C10	C12:1	C12
EZ Rich + 100mM Glycerol	Δ0763 Δ4549 + 125	0.022	0.546	0.020	0.148	0.000
	3KO + 125	0.037	0.556	0.000	0.087	0.000

Supplementary Table 6. FAME yield. FAME yield as a percent of maximum theoretical yield when grown on EZ Rich supplemented with 100 mM glycerol in 250 mL shake flask cultures.

	C6	C8	C10	C12:1	C12
FAME Theoretical yield (mol/mol of glycerol)	0.350	0.267	0.216	0.184	0.182
FAME Theoretical yield from 100mM glycerol (g/L)	4.551	4.227	4.027	3.917	3.891

Supplementary Table 7. Calculated FAME theoretical yield. Calculated FAME maximum theoretical yield from 100 mM glycerol.

Organism	Strategy	Major FFA Chain Lengths	Growth Media	Total FFA Titer (mg/L)	Ref.
Pseudomonas putida KT2440	Medium-chain fatty acid CoA ligase knockout (ΔPP_0763 ΔPP_4549), acyl-ACP thioesterase overexpression (R3.M4)	C6-C12	EZ Rich + 100 mM glycerol	189	This study
			TB	197	
			Diluted sorghum hydrolysate	561	
	Medium- and long-chain fatty acid CoA ligase knockout (3KO), acyl-ACP thioesterase overexpression (R3.M4)	C6-C16	EZ Rich + 100 mM glycerol	259	This study
			TB	671	
			Diluted sorghum hydrolysate	486	
Escherichia coli	Long-chain CoA ligase knockout, acyl-ACP thioesterase overexpression (CpFatB1 mutant)	C8	MOPS + 1% glucose	~600	[2]
			MOPS + 20 g/L glycerol, 10 g/L tryptone, 5 g/L yeast extract	~2200	
	Long-chain CoA ligase knockout, acyl-ACP thioesterase overexpression (R3.M4)	C6-C16	LB + 0.4% glycerol	~793	[3]
	r-BOX coupled with formate dehydrogenase, acetyl-CoA synthetase, phosphoenolpyruvate carboxykinase, and fatty acyl-CoA thioesterase overexpression	C6-C10	MOPS + 15 g/L glucose, 3 g/L sodium acetate, 0.5-1.5 g/L sodium formate, 10 g/L $NaHCO_3$	4700	[4]
Yarrowia lipolytica	Engineered hybrid FAS1 fused to thioesterase	C12-C18	1.7 g/L yeast nitrogen base, 60 g/L glucose, 1.32 g/L ammonium sulfate	1300	[5]
	Engineered KS domain of FAS1	C14-C18	Minimal medium + 80 g/L glucose, 1.5 g/L ammonium sulfate, oligo elements	~1340	[6]
Saccharomyces cerevisiae	Engineered KS, MPT, and AT domains of FAS	C6-C12	YPD	484	[7]
Synechocystis sp. PCC 6803	Acyl-ACP synthetase knockout, acyl-ACP thioesterase overexpression ('UcFatB1)	C12	BG11-Co + 1% CO_2 (v/v)	~55	[8]

Supplementary Table 8. FFA production strategies and titers across different host organisms.

Organism	Strategy	Major FAME Chain Lengths	Growth Media	Total FAME Titer (mg/L)	Reference
Pseudomonas putida KT2440	Medium-chain fatty acid CoA ligase knockout (ΔPP_0763 ΔPP_4549), acyl-ACP thioesterase overexpression (R3.M4), fatty acid methyltransferase overexpression (MmFAMT)	C6-C12	EZ Rich + 100 mM glycerol	31	This study
			TB	302	
			Diluted sorghum hydrolysate	63	
	Medium- and long-chain fatty acid CoA ligase knockout (3KO), acyl-ACP thioesterase overexpression (R3.M4), fatty acid methyltransferase overexpression (MmFAMT)	C6-C12	EZ Rich + 100 mM glycerol	29	This study
			TB	279	
			Diluted sorghum hydrolysate	224	
Escherichia coli	3-hydroxyacyl ACP:CoA transacylase, acyl-ACP thioesterase (tesB), methionine adenosyltransferase, and methyltransferase (MmFAMT) overexpression in MetJ repressor knockout strain	C10-C14	M9 minimal medium + 2% glucose	16	[9]
	Long-chain CoA ligase and 2-acylglycerophosphoethanolamine acyltransferase knockout, methionine adenosyl transferase, acyl-ACP thioesterase (BTE), and methyl transferase (DmJHAMT) overexpression	C12-C14	TB	560	[10]
Synechocystis sp. PCC 6803	Acyl-ACP synthetase knockout, acyl-ACP thioesterase overexpression ('UcFatB1), methyl transferase overexpression (DmJHAMT)	C12	BG11-Co + 1% CO_2 (v/v)	115	[8]

Supplementary Table 9. FAME production strategies and titers across different host organisms.

Strain	Description	JBEI part ID	Reference
E. coli XL1 Blue			Agilent
E. coli S17-1 λpir			[11]
P. putida KT2440	Wildtype		ATCC 47054
P. putida ΔPP_0763	Strain with complete internal in-frame deletion of PP_0763	JPUB_020583	This study
P. putida ΔPP_4549	Strain with complete internal in-frame deletion of PP_4549	JPUB_020584	This study
P. putida ΔPP_0763 ΔPP_4549	Double knockout strain with complete internal in-frame deletion of PP_0763 and PP_4549	JPUB_020585	This study
P. putida ΔPP_4549-50	Double knockout strain with complete internal in-frame deletion of PP_4549 and PP_4550	JPUB_020586	This study
P. putida ΔPP_0763 ΔPP_4549-50	Triple knockout strain with complete internal in-frame deletion of PP_0763, PP_4549, and PP_4550. Alias 3KO.	JPUB_020587	This study

Supplementary Table 10. Strains used in this study. All strains have been deposited in the public version of JBEI registry (http://public-registry.jbei.org) and are physically available from the corresponding author upon request.

Plasmid	Description	Origin(s)	Promoter	Selection Marker (s)	JBEI part ID	Reference
pBADT	Broad host-range arabinose inducible plasmid	pBBR1 (Broad-host-range)	araBAD (arabinose inducible)	Kanamycin		[12]
pBADT-RFP	pBADT derivative harboring RFP					[12]
113	pBADT derivative harboring wildtype ‘TesA				JPUB_020588	This study
114	pBADT derivative harboring ‘TesA variant L109P				JPUB_020589	This study
115	pBADT derivative harboring ‘TesA variant R3.M4				JPUB_020590	This study
116	pBADT derivative harboring ‘TesA variant CM-5				JPUB_020591	This study
117	pBADT derivative harboring ‘TesA variant RD-2				JPUB_020592	This study
125	pBADT derivative harboring ‘TesA variant R3.M4 and MmFAMT, a fatty acid methyltransferase from *Mycobacterium marinum*				JPUB_020593	This study
pMQ30	Suicide vector for allelic replacement of Gm^r, SacB	ColE1 (*E. coli*)<br>OriT	N/A	Gentamicin, Sucrose (counter-selection)		[13]
pMQ30 ΔPP_0763	pMQ30 derivative harboring 1-kb flanking regions of PP_0763				JPUB_020580	This study
pMQ30 ΔPP_4549	pMQ30 derivative harboring 1-kb flanking regions of PP_4549				JPUB_020582	This study
pMQ30 ΔPP_4549-50	pMQ30 derivative harboring 1-kb flanking regions of PP_4549 and PP_4550				JPUB_020581	This study

Supplementary Table 11. Plasmids used in this study. Plasmids in blue cells are parent plasmids from which the plasmids used in this study were derived. Plasmids in white cells were used in this study. All plasmids have been deposited in the public version of JBEI registry (http://public-registry.jbei.org) and are physically available from the corresponding author upon request.

Supplementary References

1. Thompson, M. G. *et al.* Fatty Acid and Alcohol Metabolism in Pseudomonas putida: Functional Analysis Using Random Barcode Transposon Sequencing. *Appl. Environ. Microbiol.* **86**, (2020).
2. Hernández Lozada, N. J. *et al.* Highly Active C 8 -Acyl-ACP Thioesterase Variant Isolated by a Synthetic Selection Strategy. *ACS Synth. Biol.* **7**, 2205–2215 (2018).
3. Grisewood, M. J. *et al.* Computational Redesign of Acyl-ACP Thioesterase with Improved Selectivity toward Medium-Chain-Length Fatty Acids. *ACS Catal.* **7**, 3837–3849 (2017).
4. Wu, J. *et al.* Improving metabolic efficiency of the reverse beta-oxidation cycle by balancing redox cofactor requirement. *Metab. Eng.* **44**, 313–324 (2017).
5. Xu, P., Qiao, K., Ahn, W. S. & Stephanopoulos, G. Engineering Yarrowia lipolytica as a platform for synthesis of drop-in transportation fuels and oleochemicals. *Proc. Natl. Acad. Sci. U. S. A.* **113**, 10848–10853 (2016).
6. Rigouin, C. *et al.* Production of Medium Chain Fatty Acids by Yarrowia lipolytica: Combining Molecular Design and TALEN to Engineer the Fatty Acid Synthase. *ACS Synth. Biol.* **6**, 1870–1879 (2017).
7. Gajewski, J., Pavlovic, R., Fischer, M., Boles, E. & Grininger, M. Engineering fungal de novo fatty acid synthesis for short chain fatty acid production. *Nat. Commun.* **8**, (2017).
8. Yunus, I. S., Palma, A., Trudeau, D. L., Tawfik, D. S. & Jones, P. R. Methanol-free biosynthesis of fatty acid methyl ester (FAME) in Synechocystis sp. PCC 6803. *Metab. Eng.* **57**, 217–227 (2020).
9. Nawabi, P., Bauer, S., Kyrpides, N. & Lykidis, A. Engineering Escherichia coli for biodiesel production utilizing a bacterial fatty acid methyltransferase. *Appl. Environ. Microbiol.* **77**, 8052–8061 (2011).
10. Sherkhanov, S., Korman, T. P., Clarke, S. G. & Bowie, J. U. Production of FAME biodiesel in E. coli by direct methylation with an insect enzyme. *Sci. Rep.* **6**, 1–10 (2016).
11. de Lorenzo, V., Cases, I., Herrero, M. & Timmis, K. N. Early and Late Responses of TOL Promoters to Pathway Inducers: Identification of Postexponential Promoters in Pseudomonas putida with lacZ-tet Bicistronic Reporters. *J. Bacteriol.* **175**, 6902–6907 (1993).
12. Bi, C. *et al.* Development of a broad-host synthetic biology toolbox for ralstonia eutropha and its application to engineering hydrocarbon biofuel production. *Microb. Cell Fact.* **12**, 1–10 (2013).
13. Shanks, R. M. Q., Caiazza, N. C., Hinsa, S. M., Toutain, C. M. & O’Toole, G. A. Saccharomyces cerevisiae-based molecular tool kit for manipulation of genes from gram-negative bacteria. *Appl. Environ. Microbiol.* **72**, 5027–5036 (2006).
