# Supplementary material for: Engineering Pseudomonas putida KT2440 for chain length tailored free fatty acid and oleochemical production
Source: Commun Biol. 2022 Dec 12;5:1363. doi: 10.1038/s42003-022-04336-2 (PMC9744835; doi:10.1038/s42003-022-04336-2)
Supplement: Supplementary file 3 — Description of Additional Supplementary Data [file 42003_2022_4336_MOESM3_ESM.docx]

**Description of Additional Supplementary Files**

**File name:** Supplementary Data 1

**Description:** Source data for Figure 2-6 and Supplementary Figure 3.
