# Supplementary material for: Engineering Pseudomonas putida KT2440 for chain length tailored free fatty acid and oleochemical production
Source: Commun Biol. 2022 Dec 12;5:1363. doi: 10.1038/s42003-022-04336-2 (PMC9744835; doi:10.1038/s42003-022-04336-2)
Supplement: Supplementary file 5 — Reporting Summary-New [file 42003_2022_4336_MOESM5_ESM.pdf]

nature portfolio

Corresponding author(s): Jay D. Keasling

Last updated by author(s): Nov 29, 2022

Reporting Summary

Nature Portfolio wishes to improve the reproducibility of the work that we publish. This form provides structure for consistency and transparency in reporting. For further information on Nature Portfolio policies, see our Editorial Policies and the Editorial Policy Checklist.

Statistics

For all statistical analyses, confirm that the following items are present in the figure legend, table legend, main text, or Methods section.

n/a	Confirmed	
☐	☒	The exact sample size (*n*) for each experimental group/condition, given as a discrete number and unit of measurement
☐	☒	A statement on whether measurements were taken from distinct samples or whether the same sample was measured repeatedly
☒	☐	The statistical test(s) used AND whether they are one- or two-sided<br>*Only common tests should be described solely by name; describe more complex techniques in the Methods section.*
☒	☐	A description of all covariates tested
☒	☐	A description of any assumptions or corrections, such as tests of normality and adjustment for multiple comparisons
☐	☒	A full description of the statistical parameters including central tendency (e.g. means) or other basic estimates (e.g. regression coefficient) AND variation (e.g. standard deviation) or associated estimates of uncertainty (e.g. confidence intervals)
☒	☐	For null hypothesis testing, the test statistic (e.g. *F*, *t*, *r*) with confidence intervals, effect sizes, degrees of freedom and *P* value noted<br>*Give P values as exact values whenever suitable.*
☒	☐	For Bayesian analysis, information on the choice of priors and Markov chain Monte Carlo settings
☒	☐	For hierarchical and complex designs, identification of the appropriate level for tests and full reporting of outcomes
☒	☐	Estimates of effect sizes (e.g. Cohen's *d*, Pearson's *r*), indicating how they were calculated

Our web collection on statistics for biologists contains articles on many of the points above.

Software and code

Policy information about availability of computer code

Data collection	GC/MS data were collected using an Agilent 6890 GC and Agilent 5973 MS running Agilent ChemStation.
Data analysis	Statistical analyses (data averaging, calculation of standard errors) were performed using custom-written Python scripts that used publicly available libraries including numpy and scipy. Chemstation Enhanced Data Analysis program was used to analyze GC/MS data. Theoretical yield calculations were conducted using the publicly available COBRApy metabolic modelling package in Python.

For manuscripts utilizing custom algorithms or software that are central to the research but not yet described in published literature, software must be made available to editors and reviewers. We strongly encourage code deposition in a community repository (e.g. GitHub). See the Nature Portfolio guidelines for submitting code & software for further information.

Data

Policy information about availability of data

All manuscripts must include a data availability statement. This statement should provide the following information, where applicable:

- Accession codes, unique identifiers, or web links for publicly available datasets
- A description of any restrictions on data availability
- For clinical datasets or third party data, please ensure that the statement adheres to our policy

Plasmids and strains used in this study are listed in Supplementary Tables 10 and 11. The plasmids and strains have been deposited in the public version of JBEI

registry (http://public-registry.jbei.org) from which physical strains can be ordered. Source data for Figure 2-6 and Supplementary Figure 3 is found in Supplementary Data 1.

Human research participants

Policy information about studies involving human research participants and Sex and Gender in Research.

Reporting on sex and gender	N/A
Population characteristics	N/A
Recruitment	N/A
Ethics oversight	N/A

Note that full information on the approval of the study protocol must also be provided in the manuscript.

Field-specific reporting

Please select the one below that is the best fit for your research. If you are not sure, read the appropriate sections before making your selection.

☒ Life sciences ☐ Behavioural & social sciences ☐ Ecological, evolutionary & environmental sciences

For a reference copy of the document with all sections, see nature.com/documents/nr-reporting-summary-flat.pdf

Life sciences study design

All studies must disclose on these points even when the disclosure is negative.

Sample size	No sample size calculation was performed. The sample size of N=3 for biologically independent replicates is standard practice in the field of metabolic engineering and has demonstrated itself to be sufficient for titer calculations.
Data exclusions	No data were excluded.
Replication	The use of defined media (EZ Rich-derived media) helps ensure reproducibility across different batches of media preparations and across different groups of researchers. Several strains and conditions were repeated in experiments across figures (e.g. FFA production by 3KO + 115 in EZ Rich + 100 mM glycerol) and yielded similar results across experiments. All attempts at replication were successful.
Randomization	All the samples tested within a figure were grown at the same time alongside each other in the incubator. The colonies used to inoculate starter cultures were randomly selected.
Blinding	Blinding was not relevant to the study because GC/MS peak area integrations were performed systematically by Chemstation Enhanced Data Analysis software and the experiments did not involve any human subjects.

Reporting for specific materials, systems and methods

We require information from authors about some types of materials, experimental systems and methods used in many studies. Here, indicate whether each material, system or method listed is relevant to your study. If you are not sure if a list item applies to your research, read the appropriate section before selecting a response.

Materials & experimental systems

n/a	Involved in the study
☒	☐ Antibodies
☒	☐ Eukaryotic cell lines
☒	☐ Palaeontology and archaeology
☒	☐ Animals and other organisms
☒	☐ Clinical data
☒	☐ Dual use research of concern

Methods

n/a	Involved in the study
☒	☐ ChIP-seq
☒	☐ Flow cytometry
☒	☐ MRI-based neuroimaging
